# How Nutritionally Sound Is Commercially Available Vegan Pet Food Relative to Meat-Based Pet Food? A Systematic Review

**DOI:** 10.3390/ani16142232

**Published:** 2026-07-18

**Authors:** Jenny L. Mace, Billy Nicholles, Andrew Knight

**Affiliations:** 1 Centre for Ethics, Philosophy and Public Affairs, University of St Andrews, St Andrews KY16 9AL, UK; jm609@st-andrews.ac.uk; 2 Mace Animal Welfare, Office 508, 18 Young Street, Unit LGE, Edinburgh EH2 4JB, UK; 3 Bryant Research, 71–75 Shelton Street, Covent Garden, London WC2H 9JQ, UK; billy@bryantresearch.co.uk; 4 School of Veterinary Medicine, College of Environmental and Life Sciences, Murdoch University, 90 South St., Murdoch, WA 6150, Australia; 5 School of Environment and Science, Griffith University, Nathan, QLD 4111, Australia; 6 Animal Welfare Research Group, Faculty of Health and Wellbeing, University of Winchester, Sparkford Road, Winchester SO22 4NR, UK; 7 Sustainable Pet Food Foundation, 147 Station Road, London E4 6AG, UK

**Keywords:** vegan pet food, meat-based pet food, vegan dog food, vegan cat food, pet nutrition

## Abstract

Single studies are often cited to support claims of nutritional soundness or unsoundness regarding vegan or meat-based pet foods when compared to nutritional guidelines from authorities such as the Association of American Feed Control Officials (AAFCO), the European Pet Food Industry Federation (FEDIAF), and the National Research Council (NRC). However, there is little overarching evidence concerning the nutritional soundness of these different pet food types. Such overarching evidence is increasingly important as alternative vegan or meat-based pet food types become more available, such as insect-based, raw meat, and microbial protein-based pet foods. In this systematic review, 54 relevant studies examining the nutritional soundness of vegan, vegetarian, or meat-based dog or cat foods were descriptively analyzed. Only 10 (18.5%) studies found *no* nutritional deficiencies or excesses across their sampled pet foods relative to AAFCO, FEDIAF, or NRC guidelines. Numerous nutrient deficiencies and excesses were found across all pet food types. Most commonly, these included deficiencies in calcium, phosphorus, and zinc for both dog and cat food, as well as the amino acid taurine exclusively in cat food. The nutrients found to be most commonly in excess were also calcium and phosphorus, and copper—with the latter breaching not only FEDIAF requirements, but also EU legislative limits. Meat-based, insect-based, veterinary prescription, and vegan pet food types each had particularly at-risk nutrients that pet food companies should be aware of. For instance, vegan pet foods often performed well in terms of their fatty acid profile and selenium levels, but some had deficiencies in certain B vitamins. In contrast, some meat-based pet foods were prone to selenium deficiencies. Generally speaking, aside from such specific nutritional flaws, problems were observed across all pet food types. In fact, dog and cat foods (including wet and dry, growth and adult maintenance) of various types commonly failed to fulfil *multiple* nutritional requirements. To safeguard dog and cat health and welfare, urgent interventions are warranted to improve the nutritional soundness of many pet foods.

## Simple Summary

Based on laboratory analyses of both vegan and meat-based pet foods, some studies have suggested pet food samples of either type to be nutritionally sound, while others have demonstrated nutritional flaws. However, a more systematic evaluation of the nutritional soundness of vegan and meat-based pet foods has been lacking. Consequently, this study sought to more comprehensively review relevant published studies to date. Overall, 54 suitable studies were located and assessed. In total, 81.5% of the studies (*n* = 44) found nutritional inadequacies in the pet foods they examined. These nutritional inadequacies spanned multiple pet food types—both vegan and meat-based, and both dog and cat food. The nutritional inadequacies concerned multiple nutrients. More testing is required to ensure that pet foods fulfil the nutritional needs of dogs and cats that they frequently claim to or are required to.

## 1. Introduction

Interest in nutritionally sound vegan pet food has risen exponentially over the last decade. The vegan pet food market was valued at nearly USD 10 billion in 2020 [1]. It rose to almost USD 27 billion in 2024 and has a forecasted value of over USD 57 billion by 2034, representing a compound annual growth rate of 7.8% [2]. Given this fast pace of growth, it is prudent to enquire about the nutritional soundness of vegan pet food, which was an aim of the present study. This would also provide guidance for guardians who may be interested in feeding their dogs and cats vegan pet food.

There are several key drivers of this rapid growth in vegan pet food. The first is the “animal lover’s paradox” [3], where dog and cat guardians and other animal advocates seek a solution to the dilemma of caring for one animal species whilst feeding them a different animal species most likely originating from intensive animal farms. Roughly 85% of UK animal farming is classed as intensive; in the USA, this figure rises to almost 100% of animal farming [4,5,6].

Second, there is increasing awareness of companion animals’ paw prints in terms of the environmental impacts created by conventional pet food. Notably, after calculating dietary energy requirements, we previously calculated that transitioning dogs and cats on to nutritionally sound vegan diets globally could save 0.3 billion hectares of land (an area larger than Mexico in the case of dogs and one larger than that of Germany in the case of cats), reduce greenhouse gases by 0.66 Gt of CO_2_ equivalents (a quantity greater than that emitted by the UK in the case of dogs and one greater than that by New Zealand in the case of cats), and spare 8.99 Gm^3^ of water (a volume greater than renewable freshwater supplies in Denmark in the case of dogs and one greater than that in Jordan in the case of cats) [7]. Third, amidst a pet obesity crisis and reduced trust in the pet food industry, many are seeking alternative options for their pets’ diets [8]. This includes 1.3% of pets being fed exclusively vegan pet food and almost 8% being fed vegan pet food intermittently [9] (p. 7). Dodd et al. [9] also found that a further 35% of pet guardians currently feeding meat-based diets were open to transitioning their pets to nutritionally sound vegan pet food, including 78% of vegan guardians. When considering more sustainable pet food options more broadly, the figure rises to between 43% and 51% of dog and cat guardians respectively being open to transitioning their pets’ diets [10,11].

At the time of writing in early 2026 there were numerous studies confirming good health outcomes in dogs and cats fed vegan diets [12,13,14,15,16,17,18,19,20,21,22]. Additionally, a systematic review of relevant studies suggested at the very least no adverse health consequences when nutritionally sound vegan pet foods are fed to dogs or cats, and maybe even some benefits [23]. Since the publication of that systematic review, there have been further studies, including a longer-term health study [24] and a clinical study focusing on vitamin D specifically [25], with none indicating health concerns when nutritionally sound vegan diets are fed to dogs or cats.

Very few contrary studies have been published. Almost 40 years ago Yamada et al. [26] subjected eight laboratory dogs to four hours daily of enforced running at 12 km/h for two weeks following six weeks of enforced rest. When compared to the four meat-based dogs, the four plant-based dogs had lower circulating free cholesterol and suffered anemia. However, this study was limited by a very small sample size and study conditions very distant from real-world conditions for companion dogs, and its results have not been confirmed by subsequent larger studies. Over thirty years ago, Leon et al. [27] observed hypokalemic episodic polymyopathy in cats fed a vegetarian diet. However, the diet fed was deficient in potassium, and cats that received the same diet supplemented with potassium did not develop hypokalemic polymyopathy. More recently, some cases of dilated cardiomyopathy (DCM) and related cardiac abnormalities were reported in association with pulses—which are often used in grain-free plant-based pet foods [28]. Detailed subsequent Food and Drug Administration (FDA) investigations were not able to establish any increased risks associated with pulses [29], and an investigation by 100 Reporters revealed that undeclared pet food industry interests appear to have influenced the case reports that initially led to these concerns [28]. However, some authors have continued to raise concerns or call for further evaluations of this issue [30,31].

In 2024, the British Veterinary Association [32] officially affirmed the healthfulness of nutritionally sound vegan diets for dogs, whilst UK Pet Food [33] did so for both dogs and cats in 2023.

Nevertheless, concerns remain for some regarding the healthfulness of commercial vegan pet foods. This is particularly the case following some studies (e.g., [34]) that have found certain commercial vegan pet foods to be deficient in nutrients at levels recommended by the European Pet Food Industry Federation (FEDIAF) and the Association of American Feed Control Officials (AAFCO). However, there are also studies (e.g., [35]) that have found conventional meat-based pet foods to likewise be nutritionally unsound. Hence, this study aimed to offer the first systematic review of the extant literature concerning the question: How nutritionally sound is commercially available vegan pet food relative to meat-based pet food?

## 2. Methodology

### 2.1. Research Design

A systematic review was chosen as the method for this study as, at the time of writing, no systematic reviews had been published focusing on the nutritional soundness of vegan and meat-based pet foods. Systematic reviews are often considered the “gold standard” in terms of the production of optimally reliable results. This is because they reduce remaining biases present in individual studies by pooling all results together to generate a clear picture of current evidence [36]. A preliminary review was conducted to ensure that there was a sufficient number of studies to warrant a systematic review and to guide the choice of the search terms.

The updated PRISMA 2020 guidelines for systematic reviews were consulted for this study and the PRISMA checklist completed [37]. Two research databases were used for this systematic review: (1) Scopus and (2) Web of Science (All Databases) as the two leading research databases [38]. The All Databases option within Web of Science integrates the Core Collection with Medline and various additional databases. Thus, this aligns with established guidance on database selection for systematic reviews, which recommends consulting at least *three* validated sources [39]. Moreover, Web of Science Core Collection, Medline, and Scopus have recently been identified as *key* databases recommended for systematic reviews [40]. Each of these resources is either multidisciplinary or focused on biomedical research. Topics and journals featured include animal nutrition. Thus, these databases are appropriate for the present research area.

These databases were searched on 14th June 2025 exclusively. As discussed by McGowan et al. [41], the same search string was used for each database, tailored to each database’s requirements. For instance, on Scopus, the search string was (without quotation marks): “(nutr* OR vitamin OR mineral OR chemical OR element) W/3 (analysis OR assay OR composition OR profile OR evaluation OR adequacy OR quality OR levels OR quantity OR concentrations OR content OR value OR estimation OR balance OR measure OR assessment OR comparison OR formulation) W/5 (pet OR dog OR cat OR feline OR canine OR puppies OR kittens OR “felix catus” OR “canis lupus familiaris”) W/3 (food OR diet OR feed)”. No other search limits were applied.

### 2.2. Selection Criteria

To be eligible for inclusion in the final shortlist of studies, studies needed to:

(1) Collect novel empirical data regarding a nutrient analysis of commercially available pet food (i.e., of a finished product available to purchase);
(2) Examine at least one nutrient;
(3) Concern dog or cat food;
(4) Concern vegan pet food or meat-based pet food (including raw; cultivated, i.e., laboratory-grown; or insect-based pet food);
(5) Be published in a peer-reviewed journal, on a pre-print platform, or as a postgraduate thesis;
(6) Be written in English or German;
(7) Use AAFCO, FEDIAF, or National Research Council (NRC) nutrition guidelines as the comparator (given that most regions of the world defer to these bodies).

**Figure 1:**
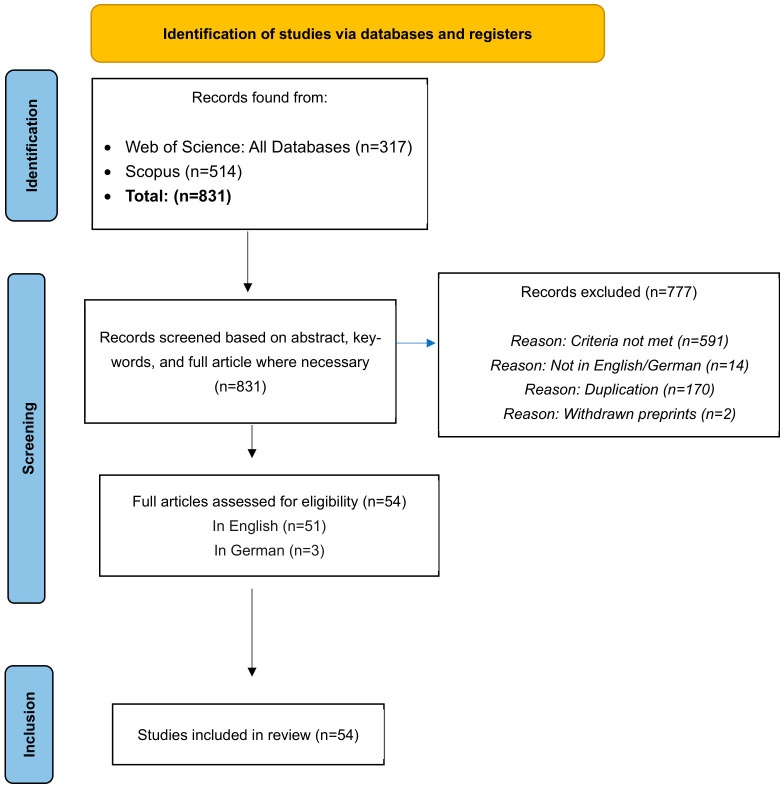
PRISMA flow diagram for the systematic review. Note: After Page et al. [42].

For a summary of the key methodological steps taken, please see Figure 1. The relevance of a study was ascertained from the title, abstract, and keywords. Where necessary, full studies were consulted. Studies focusing exclusively on nutrient analyses of new foods which were not yet commercially available were excluded, as were those focusing on the testing of new methods for nutrient analyses. Digestibility analyses were also excluded. However, if nutrient analyses were conducted prior to digestibility analyses, then the papers were still included, but only for the purpose of using the relevant data on nutritional soundness. Studies focusing on specialized diets (e.g., for senior pets) were included as these generally require good/increased nutrition, but restrictive diets (e.g., for losing weight, which may have opposite effects) were excluded. Nutritional analyses of supplements or treats were also excluded, as were studies focusing on toxins.

Only analyses of actual commercially available pet food were included (e.g., excluding analyses based solely on labelling claims). The reason for this was that it is important to see how well pet food actually meets packaging claims of being “nutritionally complete” and fulfilling FEDIAF/AAFCO/NRC requirements.

Duplicate studies resulting from the different database searches were removed. The first author created the initial shortlist. The items removed by the first co-author were checked by the second author, as were the items retained. Brief discussion resolved initial disagreements concerning eight studies. The first author explained the reasoning for the initial inclusion/exclusion of these studies, and after further consideration, agreement was reached with the second author. No additional reviewer was required.

### 2.3. Nutritional Benchmarks

The nutritional guidelines provided by AAFCO, FEDIAF, and the NRC were chosen as the nutritional comparators for assessing the nutritional soundness of pet foods as these organizations are the leading pet nutrition advisors globally and their guidelines the ones most widely used in relevant scientific studies. Table 1 and Table 2 compare each of these institutions’ nutritional guidelines for dogs and cats, respectively. AAFCO and FEDIAF regulate pet food, while the NRC conducts research and is concerned exclusively with the physiological requirements of dogs and cats [43]. AAFCO and FEDIAF minimums are thus often higher than those of the NRC as they have to consider the practical implementation of nutritional advice across a broad range of pet food types, as well as digestibility and varying bioavailability [43]. However, there are occasions when NRC amounts are higher than those of FEDIAF and AAFCO, as with sodium, chloride, and magnesium. A lack of “safe upper limits” for all three institutions can indicate inconclusive research rather than no risk from excessive intake [43]. As can be seen from Table 1 and Table 2, FEDIAF requirements are generally more comprehensive and stricter than those of AAFCO. All institutions publish updates to their nutritional guidance, the most recent being issued in 2026 for AAFCO, 2025 for FEDIAF, and 2006 for the NRC.

### 2.4. Analysis

Following O’Connor and Sargeant [36] (p. 11) and Page et al. [37], key methodological and results data were extracted from the shortlisted papers and synthesized in two “summary of findings” tables according to the extent of the nutritional deficiencies or excesses found. Only nutrients that the FEDIAF, AAFCO, or NRC list in their respective tables of nutrient requirements were considered. If a study included both prescription and non-prescription foods, only the non-prescription foods were incorporated into the present analysis. This is because some veterinary pet foods have purposefully lowered nutrient levels, such as phosphorus in the case of renal diets [47]. A meta-analysis was not performed due to the heterogeneity of the studies (and the limited resources available). For instance, a whole range of different nutrients and different numbers of nutrients were assessed across different studies. Additionally, some different methods of nutrient analyses were used. Finally, the formats that nutrients were measured in varied and were not readily convertible—such as units per amount of dry matter, per kcal of ME, and per MJ of ME. No automation tools were used, and this protocol was not pre-registered in order to retain the ability to optimally and flexibly adapt our protocol where necessary to best answer the research questions. For instance, final decisions were not made regarding the precise data to extract, or how to analyze the data, until full knowledge of data types and units could be ascertained from the final shortlist. However, no refinements to the initial plan were required; hence, all pre-analysis protocol stages outlined above remained unchanged from the original plan prior to commencing the databases searches.

### 2.5. Minimizing Bias

Steps were taken to reduce reviewer bias during both study selection and the synthesis of results, and to assess the quality and level of bias in the studies reviewed. In accordance with Boutron et al. [48], to minimize reviewer bias, the main reviewer was independent and had no conflicts of interest. Additionally, the results in a study’s abstract did not influence the decision to include or exclude a study. Information missing from some papers was marked clearly as such, and the validity and biases of each study included were assessed in a specific “evidence profile” table tailored for animal welfare, animal health, and food safety studies [36] (p. 11) to assess the “certainty of evidence” [49]. Following O’Connor and Sargeant [36], this included assessments of studies’ *risk of bias* (design considerations), *directness* (the extent to which a study answered our research question), and *risk of publication bias*. Risk of bias was assessed by primarily considering sample size, directness was assessed by considering the number of nutrients assessed, and risk of publication bias was assessed by considering sources of funding and author conflicts of interest. Assessment of the *precision* of studies’ results—such as the extent of variation in a finding, as described by Page et al. [42]—was reserved for in-text discussion where relevant; we were chiefly concerned with individual products’ results within a study’s sample.

Designed primarily for use in clinical health random controlled trials, the Grading of Recommendations Assessment, Development, and Evaluation (GRADE) tool is a systematic method for assessing the reliability of studies in a systematic review and hence the certainty of the evidence that a systematic review produces [50]. What has been described here amounts to a bounded, transparent modified GRADE (M-GRADE) approach to certainty analysis—a pragmatic approach for studies with resource limitations and/or those that are not focused on random controlled trials for healthcare interventions. We stress that only exemplar criteria in this instance were used to evaluate certainty.

### 2.6. AI Use

During the preparation of this manuscript, the authors used the large language model Perplexity (Deep Research, free plan) to paraphrase certain parts of the methodology. This was done to avoid self-plagiarism with other systematic reviews published by the authors. Perplexity was again used to paraphrase parts of the abstract for inclusion in the conclusion. The authors have reviewed and edited the output and take full responsibility for the content of this publication.

## 3. Results and Discussion

### 3.1. Essential Characteristics of Shortlisted Studies

As can be seen from the methodological steps summarized by Figure 1 and the studies summarized in Table 3 and Table 4, the searches resulted in 831 initial results. These were filtered in accordance with our criteria down to a final shortlist of 54 studies. Table 5 lists the countries represented in the studies, with USA leading. However, Europe was the continent most commonly represented, with 27 studies (50%), and all continents were represented barring Africa and Antarctica. Regardless of the specific country a study was conducted in, Table 3 and Table 4 detail that the pet food included in some studies was an international brand or a brand that could be delivered to countries other than that of its origin if ordered online. Thus, the relevance of the findings may have a broader application than the countries in which these pet food companies were based.

All of the 54 shortlisted studies were peer-reviewed published studies. Theses would have been included, but none were shortlisted. Of note is that one of the published studies was originally included as a preprint [71]; however, this study was subsequently published; thus, the preprint was replaced with the published version. Publication years spanned from 1989 to 2025, with the median being 2020. In what follows, percentages may not always total 100% due to rounding.

Five (9%) of the studies examined vegan (four) or vegetarian (one) pet foods, 49 (91%) examined meat-based pet foods (including raw meat, fish, and insect-based food), and one (2%) examined both vegan and meat-based pet foods. Dog food was the focus of 24 (44%) of the studies, cat food of 11 (20%)—including one of the vegan pet food studies—and both dog and cat food were the focus of 19 (35%) of the studies—including four of the five vegan or vegetarian pet food studies. Six (11%) of the studies focused on wet pet food, 27 (50%) on dry, 15 (28%) on both types, and six (11%) on “Other” (e.g., liquid, raw, or unclear). In terms of the nutrients examined, 36 studies (67%) focused on between six and 25 metrics, though the metrics studied ranged from one (e.g., [84]) to over 30 (e.g., [76]). Studies used recognized methods for their nutritional tests, as described by the Association of Official Analytical Chemists (AOAC International), AAFCO, and FEDIAF (e.g., see [44], pp. 33–35). Sample sizes ranged from two pet food products analyzed in a study (i.e., [99,103]) to 177 pet food products (i.e., [91]). A majority (*n* = 17, 31%) sampled between 10 and 29 pet food products.

Overall, 10 (19%) of the studies (in green in Table 3) found zero deficiencies and seven (13%) found only one deficiency—in terms of one nutrient metric—(in yellow in Table 3). It should be noted that if a study considered only one metric, then a deficiency also needed to be found in only *one* product in order to be listed (yellow) in Table 3. One additional study (2%), in blue in Table 3, found that nutrients met the minimum requirements of one nutritional body but not the other (e.g., either AAFCO or FEDIAF), though of course not all studies considered multiple nutritional bodies. These outcomes arose from the studies comparing their results to recommended minimum/maximum nutrient levels as detailed by AAFCO, FEDIAF, and/or the NRC for “complete” diets. Eighteen (33%) of the studies used the guidelines of the AAFCO, 15 (27%) used those of the FEDIAF, four (7%) used those of the NRC, and 17 (31%) used a combination of two or all three. However, the exact version (year of publication) of these institutions’ advisory documents that the studies used depended on the year studies were conducted in. There is no clear difference in the methodology of studies in Table 3 versus those in Table 4 that would explain the higher frequency of deficiencies/excesses found by studies in Table 4.

### 3.2. Certainty Analysis

Table 6 details the ultimate certainty M-GRADE for each of the 54 shortlisted studies. Two studies scored the highest M-GRADE of 5 [71,83], with two further studies scoring 4.5 [69,92]. Thus, these four studies are considered the most comprehensive and reliable from the shortlist and warrant particularly close attention. Conventional meat-based pet food, insect-based pet food, and vegan pet food featured across these studies, as well as both dog and cat food and both dry and wet food. All are in Table 4 (rather than Table 3), meaning that substantial deviations from recommendations were found. Similarly, pet foods for growth, adult, and “all” life stages also feature among these studies, as well as comparisons against both AAFCO and FEDIAF nutritional guidelines. Other studies boasted even higher sample sizes than these top four but were limited by a narrower scope of nutrient metrics. Conversely, some other studies shared a broad range of nutrient metrics akin to these top four but were weakened by very small sample sizes.

### 3.3. Deficient and Excess Nutrients

Table 7 counts the number of studies that found certain nutrients to be deficient or in excess relative to AAFCO, FEDIAF, or NRC guidelines. It is clear that deviations are commonplace across all nutrient groups. Table 7 demonstrates that deficiencies were more common than excesses. This was expected due to the dearth of guidelines regarding maximum levels for most nutrients. Calcium, phosphorus, and zinc were the nutrients most commonly deficient across both dog and cat food within the studies reviewed, in addition to taurine for cat food. Interestingly, similar nutrients also featured most commonly as *excess* nutrients for dogs (zinc and phosphorus), as well as copper too, with the latter also being the nutrient most commonly found to be in excess for cats. The FEDIAF [44] notes that its maximum levels for all microminerals are in fact *legal* maxima. Thus, EU-based studies finding excesses of microminerals mean that pet food manufacturers are contravening not only FEDIAF requirements, but also EU legislative limits (*Commission Implementing Regulation EU 2016/1095*)—for example, the zinc and manganese excesses found by Kazimierska et al. [87].

Whilst not all nutrients featured were assessed in all studies, this general pattern is reinforced when we look at the most reliable studies mentioned previously, with these same nutrients being shown as deficient or in excess across all four top-scoring studies [69,71,83,92]. There are a minority of nutrients that were never found to be deficient or in excess in either species among the shortlisted studies—namely, the amino acid histidine, vitamin B1 (thiamine), vitamin B7 (biotin), choline, and vitamin K (menadione). At least one further nutrient from each nutrient group was not found to be deficient in cat food (e.g., a few amino acids such as valine, the omega 3 fatty acid alpha-linolenic acid, and the mineral chloride), but this may be due to the lower number of studies that focused on cat food. In studies that examined both dog and cat food, dog and cat foods generally performed similarly in terms of meeting AAFCO/FEDIAF guidelines. For instance, there were examples of cat food performing worse (e.g., [60,83,85,102]) and examples of dog food performing worse (e.g., [89,96,101]).

### 3.4. Different Types of Pet Food

#### 3.4.1. Vegan Pet Food

Only six of the studies considered vegan pet food in their analyses. Four assessed vegan pet food for both dogs and cats [34,83,85,95], though it should be noted that one of the 24 samples in the study by Kanakubo et al. [95] was actually vegetarian, as it contained eggs. Another focused on vegan cat food only [99]. The sixth compared both meat-based and vegan pet food exclusively for dogs [71]. Ingredient lists were not always stated. However, most of these studies assessed *plant-based* vegan pet foods. None examined pet food based on microbial protein. The first diet based on fermented microbial protein became available in 2025 [104]. Nutritionally complete samples formulated to meet AAFCO requirements were tested for effects on feed digestibility, fecal microbiome, and health status and were found to be well tolerated in dogs by Longshaw et al. in 2025 [105]. Future research should be sure to include this novel alternative pet food option in regions where this is commercially available. The absence of any vegan pet food studies in Table 3 (studies finding no or only one deviation from nutritional requirements) is possibly a reflection of the imbalance of studies focusing on vegan pet food and the small number of articles found in Table 3. As discussed, the two studies that received the highest ratings (5) in the M-GRADE analysis (Table 6) focused on vegan pet food either solely or in combination with meat-based pet food [71,83]. Three of the others were graded 3 [34,85,99]. These were chiefly limited by their small sample sizes of two to four products. Kanakubo [95] was graded lower (2) due to measuring only a few nutrients.

The widespread nutrient deviations spanning all nutrient groups that are collectively evident (Table 4 and Table 7) persisted when considering vegan pet food specifically. Across both dogs and cats, dry and wet pet food, and pet food designed for growth or adult maintenance, recurring deviations included:

- Deficiency of crude protein;
- Deficiency of the amino acid methionine (+cystine);
- Deficiency of the fatty acid arachidonic acid;
- Deficiency of the minerals potassium and zinc;
- Imbalance of the Ca:P mineral ratio;
- Excess of the mineral copper;
- Deficiency of the vitamins A (retinol), B6 (pyridoxine), and B12 (cyanocobalamin or cobalamin).

The most notable negative difference was perhaps an overrepresentation of vitamin deficiencies. However, again mirroring the collective pattern across all 54 studies, it is of note that not all nutrients were consistently tested in the studies examining vegan pet food. Crude protein and amino acids were tested in all six of these studies (though not taurine in Starzonek et al. [85]). Crude fat and fatty acids were not tested in two studies; no minerals at all were tested in one study [95], while some minerals were not tested in others (e.g., selenium and iodine featured only in the analyses by Brociek et al. [71]); and only three of these studies considered vitamins [71,83,99].

The most notable beneficial difference between the findings of vegan pet food and the collective results (Table 7) is the absence of selenium deficiency, which ranks as the joint fifth most commonly deficient nutrient for dogs in Table 7. Selenium *excesses* may naturally be less of an issue in vegan pet food, as it is particularly found in offal. However, potential greater *deficiency* of this mineral in *meat*-based pet food could be due to a misplaced assumption of its automatic sufficiency in meat-based pet food, due to its greater presence in meat and its relative resistance to degradation when heated during processing [106].

The study by Brociek et al. [71] was graded the most highly (with the top score of 5)—jointly with Dodd et al. [83]. It was also the only study to have compared vegan and meat-based pet food. In contrast to the other vegan pet food studies, it actually found a higher average amino acid content in vegan pet food compared to meat-based pet food and no cases of crude protein deficiencies. The lowest case of crude protein occurred within a meat-based pet food. It also found significantly higher levels of unsaturated fats in the vegan pet food compared to the meat-based pet food.

#### 3.4.2. Raw Meat and Insect-Based Pet Food

No studies included cultivated meat-based pet food among their pet food samples. This also became available in 2025 [107] and should similarly be incorporated into future research in regions where it is commercially available. Other subtypes of meat-based pet food did feature among the shortlisted studies, such as insect-based pet food and raw meat-based pet food. Similar to conventional meat-based and vegan pet food, nutritional inadequacies were also prevalent within these diet types. Low calcium, phosphorus, zinc, iron, copper, selenium, various amino acids, arachidonic acid, and crude protein have been reported in insect-based foods, in addition to Ca:P imbalances and excesses of zinc, selenium, and copper [69,72,73,74,87].

No notable differences between insect- and conventional meat-based pet food were observed, apart from manganese excesses in insect-based pet food [87]. One study that featured insect-based pet food is listed in Table 3 with zero deviations from guidelines found [57]. However, this study was very narrow, only assaying crude protein and crude fat. Additionally, only one out of 36 products was insect-based. Similarly, one out of 11 products sampled in the study by Choi et al. [76] was a raw meat-based pet food, and this performed better than most other products. However, considerable deficiencies were found in raw meat-based pet food in other studies that considered this pet food type [81,82,88].

### 3.5. Welfare Hazards

The nutrient deficiencies and excesses found in these studies are important as they can adversely impact dog and cat health and welfare. As discussed, across the shortlisted studies, the most commonly deficient nutrients in both dog and cat food were calcium, phosphorus, and zinc. Calcium deficiency, for example, can lead to bone demineralization and increased fracture risk; phosphorus deficiency can lead to impaired growth and impacts on bone metabolism; and zinc deficiency can lead to emesis (vomiting), wasting and keratitis (corneal inflammation) [108,109].

As noted, across both dog and cat foods, common excesses were calcium, phosphorus, and copper. These increase the risk of skeletal abnormalities, impaired calcium uptake, and liver damage [108,110]. The amino acid taurine, required within diets by cats but not dogs, was also found to be the most commonly deficient nutrient in both wet and dry cat food. This can lead to heart disease (e.g., dilated cardiomyopathy, which can cause heart failure), retinal degeneration (causing gradual vision loss and eventual blindness), reproductive failure, immunocompromisation and other problems [108,111]. As detailed above, other common deficiencies and excesses were also found, but it was beyond the scope of this study to document all of the potential veterinary medical implications.

Both vegan and meat-based commercial diets normally include a wide range of additives and other unnatural ingredients. These could represent additional welfare hazards if they result in compromises to physical health or other determinants of animal welfare, such as the opportunity to exercise normal feeding behaviors, or the experience of a positive mental state. However, vegan diets do not appear to deviate substantially from meat-based diets in these respects. As noted in the Introduction, one systematic review [23] and multiple additional studies have not demonstrated adverse health consequences when nutritionally sound vegan pet foods are fed to dogs or cats, and in some cases limited benefits have been documented. And several studies of feeding behavior have demonstrated little difference in behavioral indicators of palatability when vegan or meat-based diets are fed. After previously analyzing behavioral indicators of palatability in 2308 dogs and 1135 cats, we concluded that “vegan pet foods are generally at least as palatable to dogs and cats as conventional meat or raw meat diets, and do not compromise their welfare, when other welfare determinants, such as nutritional requirements, are adequately provided” [112]. These results were consistent with several prior studies in this field (e.g., [113,114,115,116]).

### 3.6. Study Limitations and Further Research Suggestions

The number of studies included in the final shortlist that focused on alternative pet foods, including vegan pet foods, was substantially lower than the number focused on conventional meat-based food. This is to be expected given the predominance of conventional meat-based pet diets and the recent development of many alternative diets. In time, nutritional evaluations of alternative diets should increase.

It is also possible that certain studies may have been missed due to the exclusion of all studies not in English or German. With additional research resources a full GRADE analysis (with two raters) and a meta-analysis could be conducted using multiple researchers. The current subjective assessment of certainty based on a partial GRADE analysis—with a reliance on narrative assessment—weakens the comparative power between the studies. More subtypes of pet food could also be considered, such as hypoallergenic, senior, premium, and grain-free products. Moreover, it cannot be ruled out that some relevant studies may have been excluded due to the favoring of a more specific search string; again, this is something that could be avoided with more resources. Outside of a meta-analysis, other quantitative/statistical assessments could also be pursued, such as inter-reviewer agreement metrics (e.g., Cohen’s kappa) to further evidence the high inter-reviewer reliability.

Similarly, future research could incorporate more inclusive search criteria, for instance, not limiting studies to those explicitly comparing their nutritional analyses to AAFCO/FEDIAF/NRC standards. Other regional nutritional comparators could instead be used with standardized benchmarking performed as part of the systematic review if the data enabled this. The impact of differing nutrient metrics (e.g., whether measuring nutrients as unit per amount of dry matter, per kcal of metabolizable energy, or per MJ of metabolizable energy) and of different methods of nutrient analyses could also be considered. Finally, this study adopted a descriptive, narrative, and broad overview approach. A more fine-grained analysis could generate more detailed results—including the *extent* of deficiencies/excesses, both in terms of the extent of the deviation from guidelines and the number of products exhibiting the deficiencies/excesses *within* each shortlisted study.

### 3.7. Recommendations

The results confirm nutrient deficiencies and excesses across all studied types of pet food. An intervention in the status quo is required to rectify this, safeguard companion animal welfare, and align with consumer rights/choice—currently, pet guardians are not getting what they believe they are buying. Interventions could include more frequent compulsory product testing (with improvements mandated where necessary). This would result in better enforcement of nutritional guidelines. Countries that do not as yet have legal minimum nutritional requirements for pet food should work towards that stage as an initial step. Future research should make every effort to test *all* nutrients required by the AAFCO and FEDIAF rather than just one nutrient (or group). Additionally, nutritional composition alone does not fully determine nutritional adequacy in vivo. This can also be affected by factors such as bioavailability, digestibility, and antagonistic interactions between ingredients or their metabolites. Hence, nutritional soundness confirmation, using methods such as feeding trials and holistic health assessments, remains important. Future research should also commence nutrient assessments of the latest novel pet foods—those based on cultivated meat and microbial proteins. From a methodological perspective, a key improvement would be consistent blinding of researchers to the pet foods being tested.

## 4. Conclusions

This systematic review identified and evaluated 54 studies assessing nutrient levels within commercially available dog and cat food. Adopting a largely descriptive, narrative-based analysis, we found that 44 (81.5%) of these studies revealed nutrient deficiencies or excesses among their sampled pet foods when compared with AAFCO, FEDIAF, or NRC standards. Widespread nutrient imbalances were observed across all studied categories of pet food and all categories of nutrient. The most common deficiencies observed were calcium, phosphorus, and zinc deficiencies for both dog and cat food, as well as taurine deficiency in cat food. Nutrient excesses were less commonly observed, but typically these concerned calcium, phosphorus, and copper for both species. Different formulations, including conventional and raw meat-based, insect-based, and vegan diets, may have distinct nutrients that are particularly vulnerable to deviation from required standards, warranting careful monitoring by pet food manufacturers. For example, the vegan pet foods assessed generally performed well with respect to fatty acid composition and selenium content but were sometimes deficient in some B vitamins. Conversely, meat-based pet food often fell short of meeting recommended concentrations of selenium. Considering the observed inadequacy of both dog and cat foods (wet and dry, designed for either growth or adult maintenance, and vegan or meat-based) in achieving required nutrient profiles, immediate improvements in pet food manufacturing are warranted to protect companion animal health and welfare.

## Figures and Tables

**Table 1:** Comparison between the latest official dog nutritional requirements from FEDIAF [44], AAFCO [45], and the NRC [46] for adult maintenance, based on unit of nutrient per 100 g of dry matter and assuming an energy density of 4000 kcal ME/kg DM.

		AMOUNT ADVISED: DOG ADULT MAINTENANCE (Unit/100 g DM)		
	NUTRIENTS	FEDIAF	AAFCO	NRC
	Crude protein (g)	18.00/21.00	18.00	8.00
**Amino acids**	Arginine (g)	0.52/0.60	0.51	0.28
	Histidine (g)	0.23/0.27	0.19	0.15
	Isoleucine (g)	0.46/0.53	0.38	0.30
	Leucine (g)	0.82/0.95	0.68	0.54
	Lysine (g)	0.42/0.46	0.63	0.28
	Methionine (g)	0.40/0.46	0.33	0.26
	Methionine + cystine (g)	0.76/0.88	0.65	0.52
	Phenylalanine (g)	0.54/0.63	0.45	0.36
	Phenylalanine + tyrosine (g)	0.89/1.03	0.74	0.59
	Threonine (g)	0.52/0.60	0.48	0.34
	Tryptophan (g)	0.17/0.20	0.16	0.11
	Valine (g)	0.59/0.68	0.49	0.39
				
	Crude fat (g)	5.50	5.50	4.00 *
**Fatty acids**	Linoleic acid (omega 6) (g)	1.32/1.53	1.10	0.95 *
	Arachidonic acid (omega 6) (g)	-	-	-
	Alpha-linolenic acid (omega 3) (g)	-	-	0.036
	EPA + DHA (omega 3) (g)	-	-	0.044 *
	Omega 3:omega 6	-	*	-
				
**Macrominerals**	Calcium (g)	0.50/0.58 *	0.50 *	0.20
	Phosphorus (g)	0.40/0.46 *	0.40 *	0.30
	Ca:P	1:1 *	1:1 *	-
	Potassium (g)	0.50/0.58	0.60	0.40
	Sodium (g)	0.10/0.12	0.08	0.30 *
	Chloride (g)	0.15/0.17	0.12	1.20 *
	Magnesium (g)	0.07/0.08	0.06	0.18
**Microminerals**	Copper (mg)	0.72/0.83 **	0.73	0.60
	Iodine (mg)	0.11/0.12 **	0.10 *	0.07 *
	Iron (mg)	3.60/4.17 **	4.00	3.00
	Manganese (mg)	0.58/0.67 **	0.50	0.48
	Selenium (μg)	18.00/22.00 (d) **	35.00 *	35.00
		23.00/27.00 (w) **		
	Zinc (mg)	7.20/8.34 **	8.00	6.00
				
**Vitamins**	A, retinol (IU)	606.00/702.00 *	500.00 *	36.36 *
	D (D3: cholecalciferol) (IU)	55.20/63.90 **	50.00 *	44.00 *
	E, tocopherol (IU)	3.60/4.17	5.00	2.40
	B1, thiamine (mg)	0.21/0.25	0.23	0.18
	B2, riboflavin (mg)	0.60/0.69	0.52	0.42
	B5, pantothenic acid (mg)	1.42/1.64	1.20	1.20
	B6, pyridoxine (mg)	0.15/0.17	0.15	0.12
	B12, (cyano)cobalamin (μg)	3.35/3.87	2.80	2.80
	B3, niacin (mg)	1.64/1.89	1.36	1.36
	B9, folic acid (μg)	25.80/29.90	21.60	21.60
	B7, biotin (μg)	-	-	-
	Choline (mg)	164.00/189.00	136.00	136.00
	K, menadione (mg)	-	-	0.13

Note: Cell dashes (-) indicate no data available. Based on a sample, it seemed the majority of studies used unit per amount of dry matter (DM); hence, this measure is displayed here (unit of nutrients required per kcal of ME and per MJ of ME are also available from the institutions). Grey-shaded cells indicate “adequate” amounts rather than minimums (where no minimum is provided by the nutritional body). EPA + DHA refers to eicosapentaenoic and docosahexaenoic acid. Starred amounts (*) indicate that the institution also provides a maximum value for the nutrient. A double star (**) indicates FEDIAF’s listing of a nutrient’s maximum level as a legal requirement in the EU. The order of the listed nutrients reflects the order displayed in AAFCO/FEDIAF guidelines. Two FEDIAF amounts are provided; the first (lower) amount assumes a daily energy intake of 110 kcal/kg, while the second (higher) amount assumes a daily energy intake of 95 kcal/kg. In their published guidelines, FEDIAF presents its figures in “unit/100 g DM”, while AAFCO and the NRC display their figures in “unit/kg DM”. To enhance comparability, AAFCO’s and NRC’s figures have been homogenized with FEDIAF’s “unit/100 g DM”. Once it had been ensured that the figures were in the same unit, this simply meant dividing their “per kg DM” figures by 10 to arrive at “per 100 g DM” figures. Hence, figures may appear different to those published by the institutions. “(d)” denotes dry food and “(w)” wet food.

**Table 2:** Comparison between the latest official cat nutritional requirements from FEDIAF [44], AAFCO [45], and the NRC [46] for adult maintenance, based on unit of nutrient per 100 g of dry matter and assuming an energy density of 4000 kcal ME/kg DM.

		AMOUNT ADVISED: CAT ADULT MAINTENANCE (Unit/100 g DM)		
	NUTRIENTS	FEDIAF	AAFCO	NRC
	Crude protein (g)	25.00/33.30	26.00	16.00
**Amino acids**	Arginine (g)	1.00/1.30	1.04	0.77
	Histidine (g)	0.26/0.35	0.31	0.26
	Isoleucine (g)	0.43/0.57	0.52	0.43
	Leucine (g)	1.02/1.36	1.24	1.02
	Lysine (g)	0.34/0.45	0.83	0.27
	Methionine (g)	0.17/0.23	0.20 *	0.14
	Methionine + cystine (g)	0.34/0.45	0.40	0.27
	Phenylalanine (g)	0.40/0.53	0.42	0.40
	Phenylalanine + tyrosine (g)	1.53/2.04	1.53	1.5
	Threonine (g)	0.52/0.69	0.73	0.52
	Tryptophan (g)	0.13/0.17	0.16 *	0.13
	Valine (g)	0.51/0.68	0.62	0.51
	Taurine (g)	0.20/0.27 (w)	0.2 (w)	0.03
		0.10/0.13 (d)	0.1 (d)	
				
	Crude fat (g)	9.00	9.00	9.00 *
**Fatty acids**	Linoleic acid (omega 6) (g)	0.50/0.67	0.60	0.55 *
	Arachidonic acid (omega 6) (g)	6.00/8.00	0.02	0.002 *
	Alpha-linolenic acid (omega 3) (g)	-	-	-
	EPA + DHA (omega 3) (g)	-	-	0.01
	Omega 3:omega 6	-	-	-
				
**Macrominerals**	Calcium (g)	0.40/0.53	0.60	0.16
	Phosphorus (g)	0.26/0.35	0.50 *	0.14
	Ca:P	1:1 *	1:1	-
	Potassium (g)	0.60/0.80	0.60	0.52
	Sodium (g)	0.08/0.10	0.20	0.65 *
	Chloride (g)	0.11/0.15	0.30	0.96
	Magnesium (g)	0.04/0.05	0.04	0.20
**Microminerals**	Copper (mg)	0.50/0.67 **	0.50	0.50
	Iodine (mg)	0.13/0.17 **	0.06 *	0.13
	Iron (mg)	8.00/10.70 **	8.00	8.00
	Manganese (mg)	0.50/0.67 **	0.76	0.48
	Selenium (μg)	21.00/28.00 (d) **	30.00	30.00
		26.00/35.00 (w) **		
	Zinc (mg)	7.50/10.00 **	7.50	7.40 *
				
**Vitamins**	A, retinol (IU)	333.00/444.00 *	333.20 *	266.60 *
	D (D3: cholecalciferol) (IU)	25.00/33.30 **	28.00 *	22.40 *
	E, tocopherol (IU)	3.80/5.07	4.00	3.00
	B1, thiamine (mg)	0.44/0.59	0.56	0.44
	B2, riboflavin (mg)	0.32/0.42	0.40	0.32
	B5, pantothenic acid (mg)	0.58/0.77	0.58	0.46
	B6, pyridoxine (mg)	0.25/0.33	0.40	0.20
	B12, (cyano)cobalamin (μg)	1.76/2.35	2.00	1.80
	B3, niacin (mg)	3.20/4.21	6.00	3.20
	B9, folic acid (μg)	75.00/101.00	80.00	60.00
	B7, biotin (μg)	6.00/8.00	7.00	6.00
	Choline (mg)	240.00/320.00	240.00	204.00
	K, menadione (mg)	-	0.01	0.10

Note: Cell dashes (-) indicate no data available. A majority of studies used unit per amount of dry matter (DM); hence, this measure is displayed here (unit of nutrients required per kcal of ME and per MJ of ME are also available from the institutions). Grey-shaded cells indicate “adequate” amounts rather than minimums (where no minimum is provided by the nutritional body). EPA + DHA refers to eicosapentaenoic and docosahexaenoic acid. Starred amounts (*) indicate that the institution also provides a maximum value for the nutrient. A double star (**) indicates FEDIAF’s listing of a nutrient’s maximum level as a legal requirement in the EU. The order of the listed nutrients reflects the order displayed in AAFCO/FEDIAF guidelines. Two FEDIAF amounts are provided; the first (lower) amount assumes a daily energy intake of 110 kcal/kg, while the second (higher) amount assumes a daily energy intake of 95 kcal/kg. In their published guidelines, FEDIAF presents its figures in “unit/100 g DM”, while AAFCO and the NRC display their figures in “unit/kg DM”. To enhance comparability, AAFCO’s and NRC’s figures have been homogenized with FEDIAF’s “unit/100 g DM”. Once ensuring the figures were in the same unit, this simply meant dividing their “per kg DM” figures by 10 to arrive at “per 100 g DM” figures. Hence, figures may appear different to those published by the institutions. “(d)” denotes dry food and “(w)” wet food.

**Table 3:** Summary of 18 of the 54 shortlisted studies with the most favorable results.

	Authors	Year	Shortened Title	Country/Region	Cat/Dog	Meat, Insect, or Vegan	Dry/Wet	Nutrients Assessed	Method of Product Testing	Sample Size	Key Outcomes
1	Hamdan et al. [51]	2024	Macronutrients comparison of commercial cat foods in Malaysia	Malaysia (stores)	Cat	Meat-based	Dry	CP, Cfa	PA: AOAC	6 products	“All … products … met the [AAFCO] standard… Grade A [higher cost] cat food product has a significantly higher protein, fat, moisture, and ash content in comparison to Grade B [lower cost] cat food … Grade B has a significantly higher carbo-hydrate content as compared to Grade A cat food”.
2	Kazimierska et al. [52]	2024	The nutritional value adequacy and microbiological quality of canned foods for puppies and adult dogs	Poland (stores: local, inter-national brand)	Dog: pup, adult	Meat-based	Wet	CP	ISO 17025 methods of AOAC: CP Kjeldahl method, EE Soxhlet extraction, CF ANKOM Fiber Analyser, ME-NRC method	20 products	“[A]ll 20 canned foods presented higher protein concentrations than the recommended minimum levels … all the tested foods met the minimum standards set by FEDIAF, certain deviations were noted … for example, a reduced protein content and increased ether extract content compared to the declared values on the labels. This is especially unsafe in puppy foods”.
3	Matúš et al. [53]	2024	Fatty acid profile of commercial dry puppies’ food	Slovakia	Pup	Meat-based	Dry	FAs, fats	Cfa: reg. 2145/2004-100; FAMEs: GC system Agilent 6890A	5 products, 3 samples	“[F]our out of the five puppy … feeds … met the requirements for fatty acid content according to FEDIAF … All samples met the … minimum fat content … a minimum linoleic acid content … a minimum arachidonic acid content … and an α-linolenic acid content. Four samples met the minimum EPA + DHA content … while sample E did not meet the minimum limit”.
4	Kępińska-Pacelik et al. [54]	2023	Assessment of the content of macro-nutrients and micro-biological safety of dry dog foods	Poland: store, inter-national	Dog	Meat-based	Dry	CP, Cfa (EE)	AOAC	35 products	“In the case of macronutrients, the results in some cases were multiples of the minimum recommended levels … current nutritional guidelines do not set maximum levels for proximate components”.
5	Stercova et al. [55]	2022	Nutritional evaluation of commercial dry dog foods available on the Czech market	Czech Republic	Dog	Not stated (meat-based implied)	Dry	CP, Cfa, ×9 AAs	PA: AOAC; ME: NRC equation	60 products (only 30 for AAs)	“Most of the tested dog diets met the … recommendations. There were only a few deviations in threonine in G1 and G2 diets [low-medium cost, GI] for growing dogs … In G1 and G2, the threonine contents of most diets for growing dogs did not meet the minimum values given in the AAFCO nutrient profiles, but they met the recommendations by FEDIAF”.
6	Gizzarelli et al. [56]	2021	Clinical findings in healthy dogs fed with diets [of] different carbohydrate sources	Italy	Dog	Meat-based	Dry	CP, Cfa, ×20 AAs	Near-infrared spectroscopy, high-performance liquid chromatography	3 products	“[T]he amount of crude protein, total fat, and metabolizable energy fall into the levels recommended by FEDIAF 2020 … The amount of essential amino acids in the three tested diets were about double the minimum levels recommended by FEDIAF”.
7	Kazimierska et al. [57]	2021	Evaluation of nutritional value and microbiological safety in commercial dog food	Poland: stores	Dog	Meat-/insect-based	Dry	CP, Cfa	AOAC	36 products (27 int., 9 local brands)	“All thirty-six evaluated dry dog foods met the minimum … FEDIAF requirement for total protein and fat content … Those nutrient levels are minimum recommended allowances for commercial pet food, not minimum requirements or optimal intake levels … In that case, the tested foods compared much worse”.
8	Macías-Montes et al. [58]	2021	Nutritional evalu-ation and risk assessment of the exposure to essential and toxic elements in dogs and cats via the consumption of pelleted dry food	Spain, Gran Canaria: supermarkets, stores	Both	Meat-based	Dry	Fe, Cu, Mn, Se, Zn	ICP-MS	61 products (31 cats, 30 dogs), twice same product	“In general terms, both types of food reached 100% of the DRV for iron, copper, selenium and zinc [and manganese] … Among trace elements, selenium and manganese are above the dietary reference value, although they do not reach toxic levels … no statistically significant differences were observed regarding the content of essential elements between … premium brands vs. low-cost brands”.
9	Summers et al. [59]	2020	Evaluation of nutrient content and caloric density in commercially available foods formulated for senior cats	USA: pet food stores	Cat	Meat-based	Both	CP, Cfa, P, Ca, Mg, Na, K, D3	AOAC, CP, CF, Cfa: Dumas nitrogen combustion, Ankom filter bag technique, and acid hydrolysis, respectively. MA: ICP-OES, D3: liquid chromatography + tandem mass spectrometry	31 senior: 12 wet, 19, dry; 13 companies (cf. Summer 2020b)	“All foods for senior cats had crude protein and crude fat concentrations … phosphorus, calcium, magnesium, potassium, and sodium concentrations above the AAFCO Cat Food Nutrient Profile minimum value for adult maintenance … D3 concentrations were above the AAFCO Cat Food Nutrient Profile minimum value and below the maximum value for adult maintenance”.
10	Leiva et al. [60]	2019	Pet food quality assurance and safety and quality assur-ance survey within the Costa Rican pet food industry	Costa Rica	Both	Meat-based (inferred)	Dry	CP, Ca, P, FAs, AAs	AOAC	30 products (15 dog, 10, pup, 5 cat)—two samples	“From the diets tested … 1 sample was below the taurine limits recommended for adult cats”.
11	Burdett et al. [61]	2018	Many Canadian dog and cat foods fail to comply with the guaranteed analyses reported	Canada: sold excl. in Canada, pet retailers	Both	Meat-based (assumed)	Dry (ded-uced)	CP, Cfa, AAs (bar Met, Cys, Trp)	ISO standards, AOAC	27 products (dog, 16; cat, 11), blinded duplicate samples	“25 met or exceeded the AAFCO nutrient recommendations [meaning two did not]. Only 9 foods met all nutrient content claims listed in their guaranteed analyses. Nutrient content between species or life stages was not different … The 2 diets that failed to meet or exceed the AAFCO nutrient profiles were below the AAFCO recommendation of 1.04% for threonine”.
12	Paulelli et al. [62]	2018	Risk assessment of 22 chemical elements in dry and canned pet foods	Brazil: supermarkets, 48 brands	Both	Meat-based	Both	12 ess. mins, 10 noness. mins.	ICP-MS	88 products: dogs: 68, cats: 20; 48 brands, 3 samples	“Mean levels of all essential elements reached the minimum level recommended by AACFO. Selenium levels were very close to the maximum limit proposed by AAFCO”.
13	Kritikos et al. [63]	2018	Quantification of vitamin D3 in commercial dog foods and comparison with [AAFCO] recommendations	Canada	Dog	Meat-based (implied)	Both	D3	NRC/AAFCO (high-performance liquid chromatography–tandem mass spectrometry)	82 products	“…81 of the 82 dry and canned (wet) dog food samples evaluated contained adequate concentrations of Vit D3 as outlined in AAFCO recommendations”.
14	Pereira et al. [64]	2018	Mineral composition of dry dog foods	Portugal –stores, vets, international brands	Dog	Meat-based	Dry	Ca, P, Mg, Fe, Na, K, Se, Cu, Mn, Zn	ICP-MS, flame photometry, atomic/molecular spectrophotometry	26 products (20 adult, 6 puppy)	“With the exception of Se, all trace elements were supplied above the nutritional requirements of adult dogs. Legal limits of Cu, Se, and Zn were surpassed. The content of nonessential trace elements included values in the range of nanograms to micrograms per kg, without surpassing safe upper limits”.
15	Elias et al. [65]	2012	Neutron activation analysis for assessing chemical composition of dry dog food	Brazil	Dog	Meat-based (assumed)	Dry	Ca, Fe, K, Na, Se, Zn	NAA	34 products (pup, adult, senior)	“…the concentrations of Ca, Fe, K, Na, and Zn complied with the values required by the … AAFCO … Se concentrations varied from 0.24 to 2.14 mg kg^−1^, whereas a maximum regulatory limit has been set to 2 mg kg^−1^ … Only two samples presented values close to the maximum limit…”
16	Duran et al. [66]	2010	Trace element concentrations of some pet foods commercially available in Turkey	Turkey: markets, international	Both	Meat-based	Both	Cu, Fe, Mn	Flame atomic absorption spectrometry	15 products	“Trace metal concentrations of the investigated commercial pet food samples available in Turkey appeared to meet the regulated nutritional values by AAFCO”.
17	Williams et al. [67]	2006	Lysine content in canine diets can be severely heat damaged	Chile: stores local/inter-national brands	Dog	Meat-based (assumed)	Unclear	Lysine	Kjeldahl method, Moughan & Rutherfurd	33 products	“All of the maintenance diets and all except one of the growth diets did contain sufficient available lysine to satisfy … [AAFCO] … Although the total lysine content may be sufficient to meet lysine requirements for growth and maintenance, a large proportion of the lysine in the diets appears to have been damaged and can be expected to be unavailable to dogs”.
18	Huber et al. [68]	1991	Comparison of procedures for assessing adequacy of dog foods	USA	Dog	Not stated: meat-based implied	Dry	CP, Cfa, Ca, Cu, P, Zn	AOAC	3 products	“None of the foods exceeded the label maximal guarantee for crude fiber and moisture. Two foods …met the label minimal guarantee for protein, and the protein content of the remaining food … was within … [AAFCO] tolerances … The calcium, phosphorus, copper, and zinc content of all foods exceeded the minimal [NRC] concentrations … for growth … feeding tests provide valid assessment of pet food quality, and procedures involving only chemical analysis or calculated values may not”.

Note: Listed in order of publication year with the most recent first, followed by alphabetical order of the authors. Ten of these studies found no deviations from the AAFCO/FEDIAF/NRC guidelines (in green), seven found only one nutrient to be deficient (in yellow), and one found no deviations according to one nutritional body but did according to another (in blue). Only relevant nutrients were counted (e.g., not toxic elements). If stated, it is stipulated in the “country/region” column if the pet food(s) was sourced online or at a physical store, and if it was an international or local brand. “Pup” is shorthand for “puppy”; “CP” indicates “crude protein”; “Cfa” indicates “crude fat”. For more information on the methods of product testing, see the specific studies concerned, or for a summary see [44] (pp. 33–35).

**Table 4:** Summary of 36 of the 54 shortlisted studies that found considerable deviations from the nutritional guidelines of AAFCO, FEDIAF, and/or the NRC.

	Authors	Year	Shortened Title	Country/Region	Cat/Dog	Meat, Insect, or Vegan	Dry/Wet	Nutrients Assessed	Method of Product Testing	Sample Size	Key Outcomes
1	Baptista da Silva et al. [69]	2025	Assessment of commercial insect-based pet food products: nutrients’ concentration, labelling adequacy and determination of animal DNA	Europe	Both	Insect	Both	CP, AAs, Se, Ca, P, Ca:P, K, Mg, Cu, Fe, Zn	PA: AOAC; dry: NRC; wet: Atwater calc.; mins: ICP-OES, ISO 11885/ICP-MS, ISO 11885; AAs: RPHPLC + DAD	29 products, 4 samples, 16 brands	“Three products (10%) met FEDIAF’s recommendations, and seventeen (59%) met NRC’s recommendations. Only one (3%) adhered to both label and FEDIAF recommendations. Most nutritional inadequacies were seen in selenium, calcium, phosphorus, Ca/P ratios, and taurine”.
2	Bilgiç et al. [70]	2025	Investigation of trace and macro element contents in commercial cat foods	Turkey—made in Spain, Italy, Turkey	Cat	Meat-based (incl. fish)	Dry	Cu, Fe, Mn, Se, Zn, Mg, Ca, P	Mins: ICP-OES	96 products (51 prescr., 45 nonpres.), 4 samples	“15.5% of the [nonpresc.] group failed to provide a nutritional minimum level. 4.44% of prescription foods were below the minimum recommended Mn level … and 66% of [nonpresc.] foods were below the minimum adult maintenance value”.
3	Brociek et al. [71]	2025	Nutritional analysis of commercially available, complete plant- and meat-based dry dog foods in the UK	UK	Dog	Meat-based & vegan	Dry	CP, AAs, FAs, major/trace elements (mins.), vit. D, all B vitamins	CP: FlashEA^®^ 1112 N/Protein; AAs: uHPLC-MS/MS; FAs: mass spectrometry (GC-MS); mins.: ICP-MS; vits.: LC-MS/MS	31 products: 19 meat-based, 6 veterinary, 6 plant-based	“Nutritional composition of meat and plant-based foods were nutritionally similar, except for iodine and B-vitamins, which were lower in plant-based foods. The majority (66%) of veterinary diets with lower total protein by design, were also deficient in one or more essential amino acids. Isolated instances of non-compliance to nutritional guidelines were observed across all food-groups. Of the tested nutrients 55%, 16%, 24% and 100% of foods met all amino acid, mineral, B-vitamin, and vitamin D guidelines, respectively.”
4	Kępińska-Pacelik et al. [72]	2025	Assessment of macronutrients and fat quality in cat food	Poland: local, international	Cat	Poultry; fish; other, incl. insect	Dry	CP, FAs (SFAs, MUFAs)	PA: AOAC; FAMEs: gas chromatography + mass spectrometry	27 products	“All analyzed cat foods met the minimum recommended levels for protein and fat for adult cats … 11% of the products did not meet the minimum … level for linoleic acid, and 14% lacked arachidonic acid”.
5	Ryu et al. [73]	2025	Proximate analysis and profiles of amino acids, fatty acids, and minerals in insect-based foods for dogs	South Korea: online	Dog	Insect-based	Unclear	CP, Cfa, AAs, FAs, Ca, Mg, Zn, P, Fe, Cu, Ca-P ratio	PA: CP—automated Kjeldahl method, CF—Fibertec 8000 FOSS, Cfa—Soxtec 8000/Hydrotec 8000 FOSS, CA/DM—Organization for Standardization 6496; mins: ICP-OES; acid-stable AAs: ion exchange chromatography (Ninhydrin method); sulfur AAs: adap. AOAC; FAs: gas chromatograph	18 products, hydrolyzed	“[PA] results revealed that all 18 pet foods met the [AAFCO] guidelines … [MA] showed that while all foods met the [AAFCO] guidelines for magnesium, discrepancies were found in calcium, phosphorus, zinc, iron, and copper content, with several samples failing … 2 foods exceeded the … maximum ratio for certain fatty acids”.
6	Jacuńska et al. [74]	2024	Evaluation of the nutritional value of insect-based complete pet foods	Poland: store, vet	Dog	Insect-based	Dry, therapeutic	CP, Cfa, Ca, P	PA: AOAC; mins: atomic absorption spectrometry; F fractions: deter-gent method	14 products	“All diets were found to meet the minimum … levels from the FEDIAF nutritional guidelines for protein … and fat … In one food, there was a discrepancy of up to 19.21 g between the NDF fraction and the crude fiber content. Calcium levels were inadequate in two foods, and furthermore, twelve foods exhibited an abnormal calcium/phosphorus ratio”.
7	Or et al. [75]	2024	Evaluation of some element and mineral levels in prescription and non-prescription dog diets	Turkey—local & Euro-pean brands	Dog	Meat-based	Dry	Cu, Fe, Mn, Se, Zn, Ca, P	ICP-OES	150 products: 100 prescr./50 nonpresc.	“Some element and mineral values show significant differences between prescription and non-prescription market foods … Se was measured greater than the upper limits of FEDIAF in all food groups. P was lower than both determined minimum limits and non-prescription dry foods”.
8	Choi et al. [76]	2023	Nutritional evaluation of new types of dog foods including raw and cooked homemade-style diets	South Korea: online, local/imported	Dog	Meat-based	HM-style	CP, Cfa, 10 × mins., 11 × AAs, 5 × FAs	AOAC, gas chromatography	11 products, 3 tests per sample	“It was concluded that all foods analyzed had one or more nutrients below the AAFCO recommended levels although three to six products (26.2–54.5%) almost met the AAFCO standard except one to two criteria”.
9	Jacuńska et al. [77]	2023	Comparison of key nutrient content of commercial puppy foods with canine dietary requirements	Poland: stores/online	Pup	Meat-based	Dry	CP, Cfa, FAs	PA: AOAC; FAs: Food Industry FAME Mix, Restek	5 products (3 produc. cycles)	“The diets fulfilled nutritional requirements for protein and fat according to the FEDIAF but varied in terms of levels of essential fatty acids. Additionally, trans fat such as C18:1 elaidic acid was present in all diets”.
10	Kępińska-Pacelik et al. [78]	2023	Mineral and heavy metal content in dry dog foods with different main animal components	Poland: stores/online	Dog	Meat-based	Dry	Ca, K, Mg, Na, Fe, Mn, Zn, Cu	AOAC, colorimetry, mass spectrometry	41 products	“The worst results in terms of mineral content were obtained in mixed [animal protein] foods … pet food with a mineral composition similar to the MIN-RL may be characterized by unfavorable mineral ratios”.
11	Alborough et al. [79]	2022	Estimating short and longer-term exposure of domestic cats to dietary iodine fluctuation	UK: supermarkets, pet shops	Cats	Meat-based	Both	Iodine	ICP-MS	119 products (56 wet, 63 dry), 3 samples of each	“Of the 22% (*n* = 26 feeds) that were not compliant, the majority (*n* = 23) were below the nutritional minimum value with most (*n* = 16) being dry kibble. Iodine content of feeds did not vary considerably between types of feed or feed packaging, but variation between different batches of the same feed was 14–31% … cats are likely to experience chronically low or fluctuating dietary iodine intake”.
12	Sgorlon et al. [80]	2022	Elemental composition in commercial dry extruded and moist canned dog foods	Italy: store	Dog	Meat-based	Both	CP, Ca, P, K, Mg, Na, Ca-P ratio, Cu, Fe, Se, Mn, Zn	AOAC, ICP-OES, mass spectrometry	37 products (15 dry, 22 wet), 3 units from each	“All the analyzed foods supplied on average micro minerals above the … daily requirements of FEDIAF, except for Cu … in the MCD and Se both in DED and MCD … All the analyzed foods supplied macro elements above the … daily allowances except for K … in the DED and Mg … in MCD”.
13	Summers et al. [81]	2022	Evaluation of iron, copper and zinc concentrations in commercial foods for healthy cats	USA: grocery/pet stores	Cats	Meat-based	Both + raw	CP, Cfa, Fe, Cu, Zn	AOAC, ICP-OES	112 NP products	“13/112 food products had a mineral deficiency, of which a majority (*n* = 11/13) were raw food products”.
14	Vecchiato et al. [82]	2022	From nutritional adequacy to hygiene quality: A detailed assessment of commercial raw pet food for dogs and cats	Germany: online	Both	Meat-based	Raw	CP, Cfa, Ca, P, Zn, Cu	Weende analyses, Ca: flame emiss. spectrometry, P: modif. vanad. molybdate, Zn/Cu: atomic absorption spectr.	44 products for PA; 31 for MA	“Fat and protein content exceeded tolerated deviation from declared values in 33% and 45% of RMBDs, respectively. Each RMBD showed at least one concern regarding nutrient content. The RMBDs had high fat contents … that were negatively correlated with protein”.
15	Dodd et al. [83]	2021	Comparison of key essential nutrients in plant-based pet foods sold in Canada to American/European dietary recommendations	Canada	Both	Plant-based	Both	CP, Cfa, AAs, FAs, B12, D2, D3	Protein: Dumas method, AOAC, ICP-OES	26 products (all avail.)	“Four products met AAFCO and one product met FEDIAF nutrient recommendations for canine maintenance. No diets met AAFCO or FEDIAF recommendations for feline maintenance or growth for either species.”
16	Guidi et al. [84]	2021	Investigation on iodine levels in canine and feline canned food products in Italy	Italy: stores, local & int. brands	Both	Not stated: meat-based implied	Wet	Iodine	ICP/MS	12 products (6 dogs, 6 cats)	“…25% of the analyzed food did not respect the recommended range and … nutritional adequacy was not influenced by the distribution channel”.
17	Starzonek et al. (German) [85]	2021	Assessment of vegan complete diets for dogs and cats available in Germany	Germany	Both	Vegan	Both	CP, Cfa, Ca, K, Mg, Na, P, Zn, Cu, Mn, Fe, AAs (×8); vits. (×7)	Verbands Deutscher Landwirtschaftlicher Untersuchungs und Forschungsanstalten (VDLUFA, Method Book)	4 products (3 wet, 1 dry; 1 cat, 2 dog, 1 both)	“The supply of minerals exceeded the … allowance by 5.2- to 8.2-fold in the adult dog and by 9.0- and 11.4-fold in the adult cat. In 1 out of 4 diets, the … guidelines were undercut for [CP], [Cfa] and [P]. [Zn] and [Cu] … were in excess and/or insufficient. A shortfall … was present for [Zn] content in one diet. 2 products only met the minimum … for methionine and cystine but not the recommended … The added taurine met the recommended intake in one … diet for cats”.
18	Kara [86]	2020	Determination of the in vitro digestibility and nutrient content of commercial premium extruded foods with different types of protein content for adult dogs	Turkey	Dogs	Meat-based	Extruded (prem.)	CP	AOAC, Megazyme assay	24 products	“The CP [and EE] values of the premium type … foods … in the present study … were at the minimum [FEDIAF] recommended protein levels for the adult dog food … [S]ome commercial dog foods … had an energy content higher than the [NRC and FEDIAF] maintenance protein requirements … the typical CF content of dry pet food is 2.5% to 4.5% [acc. To NRC]. The CF contents of the F-dog foods, L-dog foods and P-dog foods from the different commercial brands in the present study ranged from 2.3% to 7.6% (av. 4.6%)”.
19	Kazimierska et al. [87]	2020	Mineral composition of cereal and cereal-free dry dog foods versus nutritional guidelines	Poland: shops	Dog	Meat-/insect-based	Dry	Ca, P, K, Na, Mg, Fe, Zn, Mn, Cu	AOAC, atomic absorption spectrometer	30 products	“All the evaluated dry dog foods met the minimum … levels for protein and fat. [18] tested dog foods (60%) did not meet at least one recommendation … Four dog foods exceeded the legal limit of Fe … five foods … of Zn; in one … Zn level was almost twice higher. Dog foods with insect protein exceeded the legal limit for Mn content. Eight dog foods had an inappropriate Ca:P ratio.”.
20	Summers et al. [88]	2020	Evaluation of phosphorus, calcium, and magnesium content in commercially available foods formulated for healthy cats	USA	Cats	Meat-based	Both + raw	CP, P, Ca, Mg, Ca:P	CP: Dumas nitrogen combustion method, MA: ICP-OES	82 products (81 with AAFCO label)	“All 81 foods [with AAFCO label] had analyzed Mg concentration above the AAFCO … minimum. The analyzed P in foods ranged from below the established AAFCO minimum … for maintenance to amounts that have been experimentally shown to cause renal dysfunction … The analyzed [CP] concentration for 3 foods was below the AAFCO … minimum value for adult maintenance”.
21	Zafalon et al. [34]	2020	Nutritional inadequacies in commercial vegan foods for dogs and cats	Brazil	Both	Vegan	Dry	Macronutrients, FAs, AAs, Ca, Cl, Cu, Fe, K, Mg, Mn, Na, P, Zn	AOAC, ISO gas chromatography, ICP-OES, high-perf. liquid chromatography	4 products (3 dogs, 1 cats)	“All foods met the minimum recommend-dations for macronutrients. All foods analyzed had one or more nutrients below the recommended levels and some presented zinc and copper excess”.
22	Brunetto et al. [89]	2019	Phosphorus and sodium contents in commercial wet foods for dogs and cats	Brazil: pet stores, internat./local br.	Both	Meat-based: implied	Wet	P, Na	AOAC spectrophotometry	25 products (13, dogs; 12, cats)	“All … contained [P] and [Na] amounts above … requirements. Three wet foods for dogs exceeded the safe … limit for [P], and four wet foods for dogs and three for cats exceeded [FEDIAF’s] 3.75 g of [Na]/1000 kcal … ME”.
23	Costa et al. [90]	2018	Evaluation of the chemical composition of dry feeds for dogs and cats	Brazil	Both	Meat-based	Dry	Ca, Cu, Fe, K, Mg, Mn, P, Zn	ICP OES	41 products (24 dog, 17 cat)	“The … concentrations ranged from 2.3 mg kg^−1^ (Ba) to 2.8 g kg^−1^ (Ca) in cat feeds, and from 3.8 mg kg^−1^ (Cu) to 3.4 g kg^−1^ (Ca) in dog feeds. The concentrations of P are in accordance with the Brazilian legislation, and Mn and Zn contents met the American legislation … [V]alues of Cu, K and Mg … in some samples were below the limits [of AAFCO]”.
24	Davies et al. [91]	2017	Mineral analysis of complete dog and cat foods in the UK and compliance with European guidelines	UK: stores	Both	Meat-based	Both	6 macronutrients, 6 major mins., 8 trace mins.	ICP-MS	177 products (97 wet, 80 dry, 48 brands; 113 cats, 64 dogs)	“A majority of foods complied with ≥8 of 11 guidelines (99% and 83% for dry and wet food, respectively), but many failed to provide nutritional minimums (e.g., Cu, 20% of wet food) or exceeded nutritional maximums (e.g., Se, 76% of wet food). Only 6% (6/97) of wet and 38% (30/80) of dry food were fully compliant. Some foods (20–30% of all analysed) had mineral imbalances, such as not having the recommended ratio of Ca:P (between 1:1 to 2:1)”.
25	Röckert et al. (German) [92]	2017	Evaluation of nutritional characteristics of commercial canned cat diets	Germany	Cat	Meat-based	Wet	CP, Cfa, Ca, P, Mg, NA, K, Cu, Mn, Se, Zn; Vits. A, E, D3; AAs (arginine, taurine)	Verbands Deutscher Landwirtschaftlic-her Untersuchungs und Forschungs-anstalten, VDLUFA	21 products	“In 12 complete foods, the [Ca] and [P] contents were threefold higher than the … requirement. In 16 of 21 complete foods, substantial discrepancies were observed between the recommendations and the analyzed trace elements … selenium contents exceeded the … requirement more than threefold. The vitamin, arginine and taurine contents showed no significant discrepancies…”.
26	Gosper et al. [93]	2016	Composition of some commercial cat foods, their package labelling, and suitability for meeting nutritional requirements	Australia: supermarket, pet stores	Cat	Meat-based (assumed)	Both	CP, FAME, AAs	AOAC	20 (10 dry, 10 wet), duplicate samples, diff. batches	“When compared with the Australian Standard … 8 did not adhere to the standards for nutrient composition. [V]arious deficiencies and excesses of [CP], [Cfa], [FA] and [AA] were observed in the majority of … foods … [as per their] satisfaction of the [AAFCO/NRC maintenance] requirements of a 4-kg adult cat”.
27	Prantil et al. [94]	2016	Nutritional analysis and microbiological evaluation of commercial enteral diets for cats	USA: vets or online	Cat	Meat-based (assumed)	Liquid	CP, Cfa, AAs, mins., thiamine (vit. B)	AOAC	7 products: only 4 complete	“None of the diets analyzed met all of the minimum nutrient concentrations”.
28	Kanakubo et al. [95]	2015	Protein and AA concentrations and labeling adequacy of commercial vegetarian diets for dogs and cats	USA: stores, online	Both	Vegan, vegetarian	Both	CP, AAs	AAFCO-listed methods plus more based on evidenced higher accuracy	24 products (13 dry, 11 wet)	“Minimum CP concentrations for the specified species and life stage were met by 23 diets; the remaining diet passed appropriate AAFCO feeding trials. Six diets did not meet all AA minimums. Of these … 1 was below … minimum requirements in 4 AAs … 2 were below in 3 AAs, 2 were below in 2 AAs, and 1 was below in 1 AA”.
29	Becker et al. (German) [96]	2013	Vitamin A excess by feeding with horse meat products containing high levels of liver	Germany	Both	Horse meat	Wet	Vit. A (retinol)	High-performance liquid chromatography	6 complete products [out of 14] (B, I, O, K, L, N)	“High amounts of vitamin A were found in some products for which liver, offal or animal by-products were labelled as contents. When feeding exclusively with one of these products, the [vit.] A supply was just below the safe upper limit for cats … [and just] above … for dogs”.
30	da Costa et al. [97]	2013	Multivariate optimization of an analytical method for the analysis of dog and cat foods by ICP OES	Brazil: supermarkets	Both	Meat-based (1 dog food “cereal flavor”)	Not stated	Vit. A (retinol)	ICP OES	10 products (5 dog, 5 cat), not stated if “complete”	“Evaluation of the elemental composition of commercial dog and cat foods showed that the macronutrients Ca, K, Mg, and P were present at levels below the [AAFCO] minimum … [L]evels of the micronutrients Cu, Fe, Mn, and Zn were in accordance with the AAFCO values”.
31	Gagné et al. [98]	2013	Evaluation of calcium, phosphorus, and selected trace mineral status in commercial dry foods for dogs	USA: stores, vets	Dog	Meat-based (assumed)	Dry	Ca, P, Zn, Fe, Cu, Mn, Se	ICP-ES/MS	50 products (45 nonprescr., 5 prescr.)	“Most (39/45) maintenance foods were in compliance with AAFCO recommendations for all mineral concentrations evaluated [meaning six were not]. [Ca] … was >7.1 g/1000 kcal of ME in 4 of 45 maintenance foods, and [P] … was >4.6 g/1000 kcal ME in 3 of these; 2 maintenance foods contained <34 mg of [Zn]/1000 kcal ME. These … were not within AAFCO … ranges. [The CA:P] ratio in foods … for dogs with renal disease was above, and [Cu] … in foods … for dogs with hepatic disease was below … [AAFCO] ranges”.
32	Gray [99]	2004	Nutritional adequacy of two vegan diets for cats	USA	Cat	Vegan	Wet, DIY mix	CP, Cfa, AAs (15), mins. (7); only nutrients likely a problem in veg* diets.	Indep. lab: Eurofins Woodson-Tenent Laboratories Division, Memphis, Tenn	2 products, only one sample	“Both diets had multiple nutrient deficiencies. … Diet B was low in protein (62 g/1000 kcal) … some amino acids were … less than … AAFCO adult minimum[s]. Both diets [had] less than the … minimum[s] for methionine and taurine. Diet A … was low in arginine and lysine”.
33	Hegedus et al. [100]	1998	Assessment of nutritional adequacy of the protein in dog foods	Hungary—stores	Dog	Meat-based	Dry	CP, AAs (18)	Ion-exchange chromatography and others	8 products, 3 samples	Quite a few AAs below required levels.
34	Brown [101]	1997	A comparison of certified and noncertified pet foods	Canada: stores	Both	Meat-based	Dry	11 macronutrients, 20 micronutrients	AOAC as per the Pet Food Certification Protocol of the Canadian Veterinary Medical Association (CVMA)	6 products (3 dog, 3 cat), 4 samples	“Both certified and noncertified products met the nutritional requirements on a consistent basis, although 1 of the noncertified dog foods consistently failed to meet the Zn requirements. [It] also failed to meet the [CVMA] standards for concentrations of protein, [Ca], and [P]. One … noncertified cat food failed to meet the … [Ca] level”.
35	Heanes [102]	1990	Vitamin A concentrations in commercial foods for dogs and cats	Australia: wholesale distributor	Both	Meat-based	Both	Vit. A	Reverse-phase high-perf. liquid chromatography with fluorometr. detec.	89 products (38 dogs, 51 cats), duplicate samples	“8% of the dog foods and 14% of the cat foods had concentrations of vitamin A below the minimum recommended 1.1 mg/kg dry matter (dm) for dogs and 1.8 mg/kg dm for pregnant or lactating cats. Canned and fish-labelled cat foods were the only varieties with less than the minimum recommended concentration of vitamin A, of which 71% were the same brand”.
36	Czarnecki-Maulden et al. [103]	1989	Evaluation of practical dry dog foods suitable for all life stages	USA	Dog	Meat-based	Dry	CP, Cfa, MA (12), VA (12)	Not stated	2 products	“Diet 1 was marginal in tryptophan concentration, and both diets contained low levels of vitamin A, but contained excesses of most of the other essential amino acids, vitamins, and minerals”.

Note: Listed in order of publication year with most recent first, followed by alphabetical order of the authors. Only relevant nutrients were counted (e.g., not toxic elements). If stated, it is stipulated in the “country/region” column if the pet food(s) was sourced online or at a physical store, and if it was an international or local brand. “Pup” is shorthand for “puppy”; “CP” indicates “crude protein”; “Cfa” indicates “crude fat”; “veg*” refers to vegetarian or vegan. For more information on the methods of product testing, see the specific studies concerned, or for a summary see [44] (pp. 33–35).

**Table 5:** Country setting of the shortlisted studies.

Country	Number of Studies
USA	9
Poland	8
Brazil	6
Canada	4
Turkey	4
Germany	4
Italy	3
UK	3
Australia	2
South Korea	2
Chile	1
Czech Republic	1
Costa Rica	1
Malaysia	1
Hungary	1
Spain	1
Portugal	1
Slovakia	1
Undisclosed	1
Total	54

**Table 6:** Modified GRADE reliability analysis for the 54 studies shortlisted using exemplar evaluation criteria.

Authors	Year	GRADE	Directness	Design Considerations	Risk of Publication Bias
Brociek et al. [71]	2025	5	Very high	25 (*n* = 19 meat-based, *n* = 6 plant-based), very detailed methodology	Minimal (two coauthors but not prior to testing or authors not involved in testing)
Dodd et al. [83]	2021	5	Very high	26 products (maximum available)	Numerous declared potential CoIs, but just university funding for this study
Baptista da Silva et al. [69]	2025	4.5	Very high	29 products, 4 samples, 16 brands, online	None
Röckert et al. [92]	2017	4.5	Very high	21 products	None
Leiva et al. [60]	2019	4	High (only few mins.)	30 (15 dog, 10, puppy, 5 cat), 2 samples/prod. batch (acc. to AAFCO)	None
Ryu et al. [73]	2025	4	Very high	18 products	None
Jacuńska et al. [74]	2024	4	Very high	14 products	None
Choi et al. [76]	2023	4	Very high	11 products, 3 tests per sample	None
Vecchiato et al. [82]	2022	4	High (only few mins.)	44 products for PA; 31 for MA	None
Sgorlon et al. [80]	2022	4	High (mins. only)	37 products (15 dry, 22 wet), 3 units from each	None
Davies et al. [91]	2017	4	High (mins. only)	177 products (97 wet, 80 dry, 48 brands)	None
Kępińska-Pacelik et al. [78]	2023	4	High (mins. only)	41 products	None
Costa et al. [90]	2018	4	High (mins. only)	41 products (24 dog, 17 cat)	None
Bilgiç et al. [70]	2025	4	High (mins. only)	96 products	None
Or et al. [75]	2024	4	High (mins. only)	150 products	None
Kępińska-Pacelik et al. [72]	2025	4	High (only macronutr.)	27 most popular products in pet stores, used AOAC methods	None
Kazimierska et al. [52]	2024	4	High (limited mins.)	20 products	None
Kazimierska et al. [87]	2020	4	High (mins. only)	30 products	None
Elias et al. [65]	2012	4	High (mins. only)	34 products (puppy, adult, senior)	None
Paulelli et al. [62]	2018	4	High (mins. only)	88 products (dogs 68, cats 20, most dry, 48 brands), triplicate samples	None
Stercova et al. [55]	2022	3.5	High (no mins.)	60 products (only 30 for AAs)	None
da Costa et al. [97]	2013	3.5	High	10 products (5 dog, 5 cat)	None
Gagné et al. [98]	2013	3.5	Med-high	45 products	None
Summers et al. [88]	2020	3.5	Med-high	31 senior products	None
Summers et al. [59]	2020	3.5	Med-high	82 products	None
Macías-Montes et al. [58]	2021	3.5	Med (only few mins.)	61 products (31 cats, 30 dogs), twice same product	None
Burdett et al. [61]	2018	3.5	Med (no mins.)	27 products (dog, 16; cat, 11), blinded duplicate samples	Royal Canin lab used: no statements on CoI/funding
Gosper et al. [93]	2016	3.5	Med (no mins.)	20 (10 dry, 10 wet), duplicate samples, different batches	None
Zafalon et al. [34]	2020	3	Very high	4 products (3 for dogs, 1 for cats: the maximum available)	None
Pereira et al. [64]	2018	3	Very high	26 products (20 adult, 6 puppy)	No CoI, but Funded by Project MinDog, which is funded by SORGAL (meat-based pet food company)
Prantil et al. [94]	2016	3	High	4 “complete” products (enteral diet)	Supported by VCA Antech, Inc—veterinary focus, but owned by Mars PetCare
Gray et al. [99]	2004	3	High	2 products, only one sample, indep. blinded anal.	None
Brown [101]	1997	3	High	6 products (3 dog, 3 cat), four samples	None
Summers et al. [81]	2022	3	Medium (only few mins.)	112 products	Authors work for Nestle Purina Pet Care, Royal Canin, Boehringer Ingelheim (animal Healthcare products), Hills, PetCo. Supplies
Hamdan et al. [51]	2024	3	Medium	6 products	Funding from Supercat International Sdn. Bhd. (sells cat food)
Hegedüs et al. [100]	1998	3	Medium	8 products, three samples	None
Huber et al. [68]	1991	3	Med.–high	3 products	None
Kazimierska et al. [57]	2021	3	Low (no mins.)	36 products (27 int., 9 local brands)	None
Kara [86]	2020	3	Low (no mins.)	24 products	None
Starzonek et al. [85]	2021	3	Very high	4 products (3 wet, 1 dry; 1 cat, 2 dog, 1 both)	None
Duran et al. [66]	2010	2.5	Med.–low (only few meas.)	15 products	None
Heanes [102]	1990	2.5	Low (few meas.)	89 products (38 dogs, 51 cats), duplicate	Advice from Arnott-Harper Pty Ltd. (pet food manuf.)
Czarnecki-Maulden et al. [103]	1989	2	Very high	2 products	Funded by Pet Food Institute (members are pet food makers)
Jacuńska et al. [77]	2023	2	Med (only PA, FAs)	5 products (3 batches for each)	None
Kanakubo et al. [95]	2015	2	Med-low (only few meas.)	24 products (13 dry, 11 wet)	None
Kępińska-Pacelik et al. [54]	2023	2	Med-low (main: microbiol. safety & PA)	35 products	None
Matúš et al. [53]	2024	2	Medium	5 products, 3 samples	Not stated
Williams et al. [67]	2006	2	Low (only lysine)	33 products	None
Brunetto et al. [89]	2019	2	Low (only 2 meas.)	25 (13, dogs; 12, cats), duplicate analyses + more if over 5% difference	None
Kritikos et al. [63]	2018	2	Low (only 1 meas.)	82 products	Royal Canin SAS funding and multiple authors paid employees of Royal Canin
Alborough et al. [79]	2022	2	Low (broader remit, only iodine)	119 products (56 wet, 63 dry), 3 samples of each	Funding from Dechra (sells cat food), though no involvement
Gizzarelli et al. [56]	2021	2	Low (broad remit, no mins.)	3 products (only a trial)	Support from Farmina Pet Food
Guidi et al. [84]	2021	2	Low (only iodine)	12 products (dogs, cats)	None
Becker et al. [96]	2013	1.5	Low (only vit. A)	6 “complete” products	None

Note: The ultimate GRADE is based on the subjective assessment of one author in consideration of the stipulated three key exemplar evaluation criteria. A value of “5” is the highest ranking, indicating high certainty of a study’s findings; “1” is the lowest ranking, indicating very low certainty of a study’s findings. “CoI” means “conflict of interest”.

**Table 7:** Number of studies showing nutrient deficiencies or excesses when compared to FEDIAF, AAFCO, or NRC requirements.

		DOG		CAT	
	NUTRIENTS	Studies Showing Deficiencies	Studies Showing Excesses	Studies Showing Deficiencies	Studies Showing Excesses
	Crude protein	4	N/A	6	N/A
**Amino acids**	Arginine	1	N/A	2	0 ^G&R^
	Histidine	0	N/A	0	N/A
	Isoleucine	3	N/A	0	N/A
	Leucine	2	N/A	1	N/A
	Lysine	4	0 ^G&R^	2	N/A
	Methionine	5	N/A	3	1
	Methionine + cystine	3	N/A	3	N/A
	Phenylalanine	3	N/A	0	N/A
	Phenylalanine + tyrosine	2	N/A	1	N/A
	Threonine	6	N/A	0	N/A
	Tryptophan	4	N/A	1	0
	Valine	2	N/A	0	N/A
	Taurine	-	-	5 (d)	N/A
				4 (w)	N/A
	
	Crude fat	1	0 ^NRC^	2	1 ^NRC^
**Fatty acids**	Linoleic acid (omega 6)	1	0 ^G&R^	2	1 ^NRC^
	Arachidonic acid (omega 6)	2 ^G&R^	N/A	3	0 ^NRC^
	Alpha-linolenic acid (omega 3)	1	N/A	0	N/A
	EPA + DHA (omega 3)	6	0 ^NRC^	1	N/A
	Omega 3:omega 6	0	2	0	N/A
	
**Macrominerals**	Calcium (Ca)	15	3	7	N/A
	Phosphorus (P)	11	5	8	1
	Ca:P	9	7	5	1
	Potassium (K)	8	N/A	5	N/A
	Sodium (Na)	3	1 ^NRC^	2	1 ^NRC^
	Chloride	2	0 ^NRC^	0	N/A
	Magnesium (Mg)	4	N/A	4	N/A
**Microminerals**	Copper (Cu)	8	7 *	4	4 *
	Iodine (I)	2	1 *	2	1 *
	Iron (Fe)	3	5 *	3	1 *
	Manganese (Mn)	3	0 *	1	0 *
	Selenium (Se)	8 (d)	3 *	2 (d)	2 *
		1	1 *	0	1 *
	Zinc (Zn)	12	7 *	7	2 *
					
**Vitamins**	A, retinol	3	1	3	1
	D (D3: cholecalciferol)	2	*	1	1 *
	E, tocopherol	0	N/A	1	N/A
	B1, thiamine	0	N/A	0	N/A
	B2, riboflavin	1	N/A	0	N/A
	B5, pantothenic acid	1	N/A	0	N/A
	B6, pyridoxine	1	N/A	1	N/A
	B12, (cyano)cobalamin	2	N/A	2	N/A
	B3, niacin	1	N/A	1	N/A
	B9, folic acid	1	N/A	0	N/A
	B7, biotin	0	N/A	0	N/A
	Choline	0	N/A	0	N/A
	K, menadione	0	N/A	0	N/A

Note: Cells with superscript “G&R” indicate that the excess requirement only pertains to diets for growth/reproduction (not for adult maintenance). “(d)” and “(w)” denote dry or wet food, respectively. Starred excesses (*) indicate nutrients that FEDIAF [44] lists as having legal maximum levels in the EU. Cells with superscript “NRC” indicate that the requirement is only stipulated by the NRC (not the FEDIAF or AAFCO). Counts do not indicate the *extent* of deficiencies/excesses, neither in terms of extent of deviation from guidelines nor the number of products exhibiting the deficiencies/excesses (just the number of studies). Not all nutrients featured in studies in comparable frequencies. The order of nutrients reflects the order displayed in AAFCO/FEDIAF guidelines.

## Data Availability

No new data were created in this study. Data sharing is not applicable to this article.
